# Mineral and Bone Disorders After Kidney Transplantation

**DOI:** 10.3389/fmed.2018.00211

**Published:** 2018-07-31

**Authors:** Chandan Vangala, Jenny Pan, Ronald T. Cotton, Venkat Ramanathan

**Affiliations:** ^1^Division of Nephrology and Solid-Organ Transplantation, Michael E. DeBakey VA Medical Center, Houston, TX, United States; ^2^Division of Abdominal Transplantation, Department of Surgery, Baylor College of Medicine, Houston, TX, United States

**Keywords:** kidney transplant, mineral and bone disorder, mineral and bone metabolism, osteoporosis, osteodystrophy, post-transplant, fracture

## Abstract

The risk of mineral and bone disorders among patients with chronic kidney disease is substantially elevated, owing largely to alterations in calcium, phosphorus, vitamin D, parathyroid hormone, and fibroblast growth factor 23. The interwoven relationship among these minerals and hormones results in maladaptive responses that are differentially affected by the process of kidney transplantation. Interpretation of conventional labs, imaging, and other fracture risk assessment tools are not standardized in the post-transplant setting. Post-transplant bone disease is not uniformly improved and considerable variation exists in monitoring and treatment practices. A spectrum of abnormalities such as hypophosphatemia, hypercalcemia, hyperparathyroidism, osteomalacia, osteopenia, and osteoporosis are commonly encountered in the post-transplant period. Thus, reducing fracture risk and other bone-related complications requires recognition of these abnormalities along with the risk incurred by concomitant immunosuppression use. As kidney transplant recipients continue to age, the drivers of bone disease vary throughout the post-transplant period among persistent hyperparathyroidism, *de novo* hyperparathyroidism, and osteoporosis. The use of anti-resorptive therapies require understanding of different options and the clinical scenarios that warrant their use. With limited studies underscoring clinical events such as fractures, expert understanding of MBD physiology, and surrogate marker interpretation is needed to determine ideal and individualized therapy.

## Introduction

Kidney transplant recipients (KTRs) are a unique population with substantial risk factors for bone disease and associated complications. KTRs can experience persistent alterations in mineral and bone disease (MBD) parameters, immunosuppressive modification of osteoblast/osteoclast activity, de-novo MBD abnormalities, and age-related bone loss. Understanding the patterns and pathophysiology of MBD pre- and post-transplant is important to accurately interpret laboratory and radiologic tests, and to guide therapy to reduce complications, including fractures.

The abrupt change in kidney function and the medications required to maintain a graft are competing forces that simultaneously affect bone disease. In the first 12 months after kidney transplantation, bone loss, as evidenced by surrogate laboratory and imaging tests, is most pronounced (1, 2). Depending on kidney function, both bone mineral density and markers of bone turnover may return to pre-transplant and even normal levels (3). Despite this eventual normalization, the risk of hip fractures during the first 3 years after kidney transplantation is greater than the already elevated hip fracture risk of dialysis-dependent patients (4, 5). Subsequently, the risk improves and is identical to that noted in pre-transplant patients. This fluctuating risk is likely related to bone loss from transplant medications and the slow evolution of MBD that accompanies near normal kidney function. Between 1997 and 2010, the incidence of hip fractures, the most substantial bone-related clinical event, was reported to be 3.8 per 1000 patient-years (6). Recent studies have demonstrated that hip fracture incidence has declined (6, 7), possibly coinciding with more steroid-minimizing or sparing protocols.

As per the Scientific Registry of Transplant Recipients (SRTR) annual data report (8), kidney waitlisted patients in year 2016, when compared to 2006, had higher percentage of patients aged ≥65 years (22.5 vs. 15.3%) and similarly, 18.4% of KTRs in 2016 were ≥65 years of age compared to around 10% in 2004. Thus, even if MBD and glucocorticoid use do not result in significant bone pathology, patients are still at risk for age-related osteopenia, and osteoporosis, especially in this era of improved patient and graft survival post-transplant.

Due to the numerous factors impacting mineral and bone homeostasis, evaluation, and treatment are not standardized. Responses to decreased bone mineral density, hyperparathyroidism, perioperative hypophosphatemia, and vitamin D disorders vary by institution. Consequently, examining treatment regimens to address mineral disorders and modify risk in bone-related disease, and the evidence that supports these plans, can help identify gaps in knowledge in need of further examination.

## MBD physiology: pre- and post-transplantation

Calcium, phosphate, vitamin D, parathyroid hormone (PTH), and bone-derived fibroblast growth factor (FGF-23) interact through a series of underlying feedback mechanisms. In patients with normal kidney function, the main goal of this interplay is to preserve serum concentrations of calcium and phosphate, and ultimately bone health. PTH synthesis and secretion is influenced by calcium, phosphate and vitamin D levels. In a functioning kidney, 25-hydroxy vitamin D (25(OH)D) is converted to active 1,25-dihydroxyvitamin D (1,25(OH)D) by 1α-hydroxylase, an enzyme that is upregulated by PTH and inhibited by high FGF-23 levels. Active vitamin D can stimulate gut calcium and phosphate absorption. Either through increased sodium-phosphate cotransporter (NaPi-IIb) expression or increased transport via brush-border membrane vesicles, vitamin D stimulates intestinal phosphate absorption (9). PTH secretion is very sensitive to its primary stimulus, serum calcium levels, and is also responsive to hyperphosphatemia (10). In conjunction with another phosphaturic hormone, FGF-23, serum phosphate levels can be restored, provided adequate kidney function. The majority of filtered phosphate is reabsorbed in the proximal tubule via the NaPi-IIa and NaPi-IIc cotransporters (11). PTH and FGF-23 (after binding to the FGF receptor-Klotho complex) interfere with tubular phosphate reabsorption by internalization of NaPi-IIa and NaPi-IIc transporters (12, 13). Absence of theses cotransporters leads to significant urinary phosphate loss. In addition, elevated FGF-23 can reduce the production of active vitamin D and interfere with transepithelial absorption of phosphorus in the intestines (14). However, paracellular gut phosphate absorption remains constant and the contributions of each mechanism of absorption are still uncertain. Lastly, PTH releases calcium and phosphorus from the bones.

In patients with chronic kidney disease (CKD) or acute kidney injury (AKI), when glomerular filtration rate (GFR) falls to less than 60 ml/min, a rise in FGF-23 production by osteocytes and osteoblasts is one of the earliest changes (15). In these patients, serum phosphate is either normal or even slightly lower due to high FGF-23 levels. The exact stimulus for FGF-23 production in the bone is not certain, but 1,25(OH)D, dietary phosphate, and reduced clearance have been reported as potential mechanisms of elevated serum levels (16–19).

The distal convoluted tubule is responsible for the majority of α-klotho production (20). Klotho has been deemed an antifibrotic, antioxidant, and anti-aging protein. Within the domain of MBD, membrane-bound α-klotho has a well-defined role in forming a complex with its obligate co-receptor, FGF-23, and increasing phosphaturia. However, all of the endocrine functions of soluble α-klotho independent of FGF-23 have not been fully uncovered. In addition to the kidney, α-klotho is produced by chief cells of the parathyroid gland (21). With FGF receptors also expressed in the parathyroid gland, speculation exists on whether α-klotho in conjunction with FGF-23 may decrease PTH production, possibly by increasing expression of both calcium-sensing receptors and vitamin D receptors (21, 22). Yet, despite increasing FGF-23 levels in CKD, hyperparathyroidism persists due to other stimuli.

**Figure 1:**
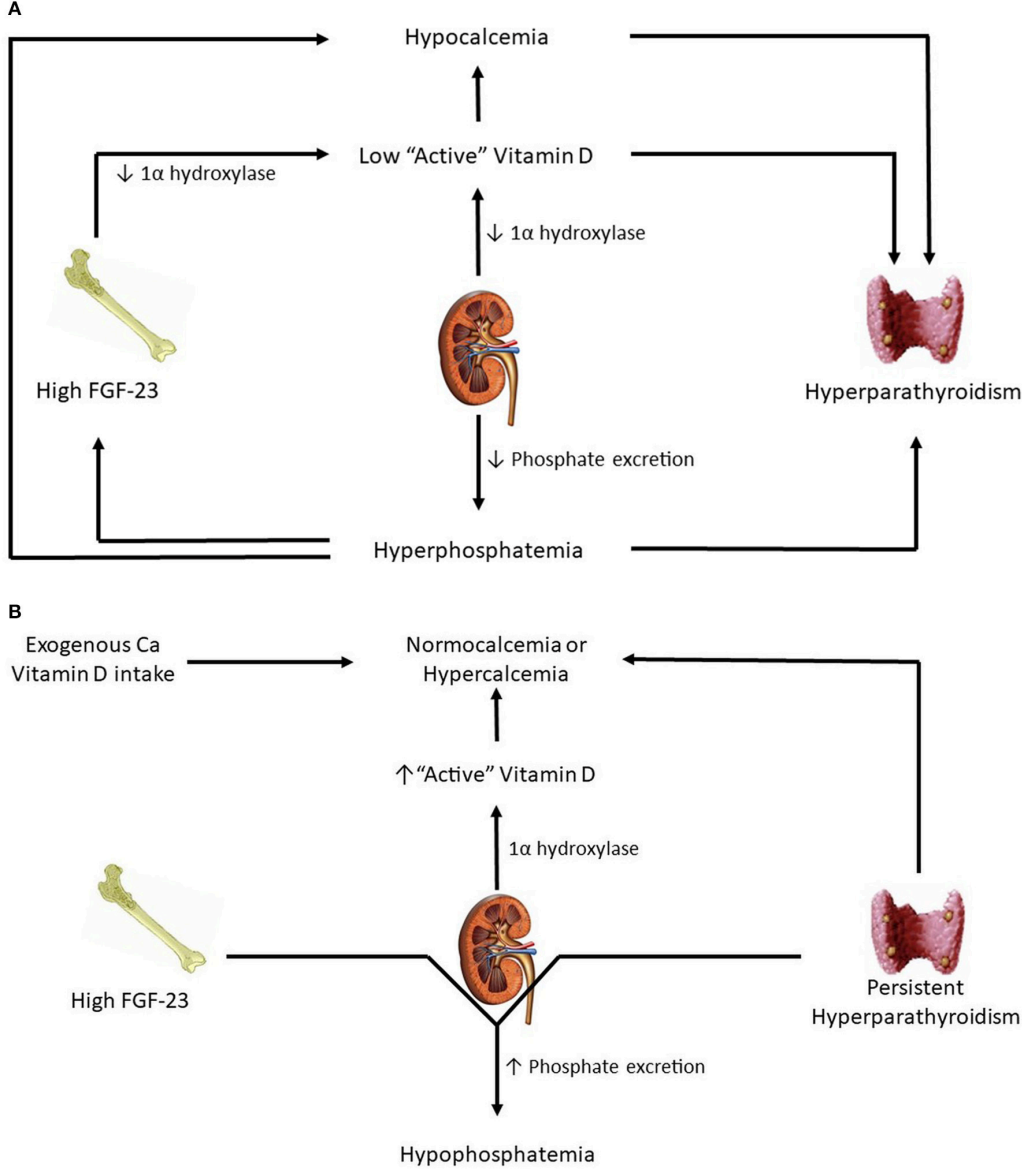
**(A)** Patients in advanced kidney disease. **(B)** Changes in kidney transplant patients with good allograft function.

As CKD advances and GFR falls further, phosphate excretion must increase per nephron to maintain balance. For reasons that have yet to be elucidated, FGF-23 levels increase. A combination of high FGF-23 levels and reduced functional nephron mass leads to low 1α-hydroxylase activity and resultant low active vitamin D levels. These lower active vitamin D levels, in conjunction with resulting hypocalcemia, stimulate a subsequent increase in PTH (15). Eventually, once the renal capacity is critically reduced, phosphorus retention and hyperphosphatemia result. Hyperphosphatemia stimulates even more PTH secretion, perpetuating further a maladaptive response. Of note, confusion exists over what PTH levels are deleterious because skeletal resistance to PTH has been recognized in patients with kidney disease(23). Overall, the end result of progressive CKD is the development of hyperparathyroidism, hypovitaminosis D, and hypocalcemia (Figure 1A).

Immediately post-transplant, circulating levels of FGF-23 and PTH are still high. However, with successful transplantation, GFR is significantly higher. As a result, high levels of PTH and FGF-23 result in significant urinary phosphorus losses and hypophosphatemia (Figure 1B). FGF-23 levels drop dramatically at 3 months post-transplantation (24) and at 1 year mimic levels seen in CKD patients with comparable eGFR (25). From a vitamin D perspective, a functioning transplanted kidney and eventual reductions in FGF-23 lead to a gradual increase in hydroxylation of 25(OH)D to active 1,25(OH)D, thereby leading to higher gut absorption of calcium and phosphorus. How α-klotho levels change in the transplant setting is not yet clear. Smaller studies have reported substantial increases in urinary soluble klotho on postoperative day 2 and 5 while no substantial distinction has been detected in serum levels before and after transplant (26, 27).

Understanding of MBD physiology is not yet complete. While the role of FGF-23 in phosphate and vitamin D metabolism has been increasingly uncovered, uncertainty over its most proximal stimulus remains. Several studies have demonstrated increases in FGF-23 with phosphate loading (28–31) and reduced levels with dietary restriction and binders (32); yet, serum phosphate levels when altered by intravenous administration have not yielded changes in FGF-23 (30). Clearly, the mechanism of FGF-23 stimulation lies within phosphate homeostasis but may not be as direct as an inverse relationship with serum phosphate levels. Limited understanding exists about the role of membrane-bound α-klotho to modulate FGF-23 signal transduction on other target organs besides the kidney. Additionally, further inquiry into soluble α-klotho's capacity as an endocrine factor is needed.

## Clinical spectrum

Clinical disorders related to MBD in KTRs include calcium and phosphate disorders, vitamin D deficiency, secondary and tertiary hyperparathyroidism, osteoporosis, and osteonecrosis (Table 1). While there are other MBD disorders including vascular calcification and kidney stones, this review will focus on common mineral and bone clinical disorders following kidney transplantation.

**Table 1:** Mineral and bone disorders after kidney transplantation.

Calcium Disorders
Hypercalcemia
*Hyperparathyroidism*
*Exogenous intake of vitamin D and Calcium*
Hypocalcemia
*Parathyroidectomy: Hungry Bone disease*
Phosphorus Disorders
Hypophosphatemia
*High FGF-23 and PTH*
*Drugs: Steroids, Sirolimus, Tenofovir, Ferric carboxy-maltose*
Hyperphosphatemia
*Delayed graft function or CKD in allograft*
Vitamin D Disorders
Hypovitaminosis D
PTH Disorders
Hyperparathyroidism
*Polyclonal or monoclonal hyperplasia*
*CKD in allograft*
Osteopenia and Osteoporosis
Aging
Residual MBD
*Hyperparathyroidism*
Hypogonadism
Medications
*Glucocorticoids, proton pump inhibitors, calcineurin inhibitors*
Osteonecrosis
Glucocorticoids

### Calcium disorders

#### Hypercalcemia

Hypercalcemia is common after kidney transplantation and has been reported in 11–31% of KTRs within 1 year (33–38). In some studies, prevalence of hypercalcemia is noted in more than 50% of patients, especially in the subset of patients who had moderate to severe hyperparathyroidism prior to kidney transplantation. Severe hypercalcemia can cause acute kidney injury in the allograft due to volume contraction and by reducing perfusion to the allograft by direct vasoconstriction. In the presence of high PTH, phosphaturia and alkaline urine resulting from concurrent use of oral alkali, there is also concern of nephrocalcinosis.

The primary drivers of hypercalcemia are persistent hyperparathyroidism and high vitamin D levels. In addition to high PTH levels, resolution of uremia post-transplant is also associated with a decrease in skeletal resistance to PTH. In addition to these endogenous changes, most transplant programs use calcium and vitamin D supplements, especially when a steroid is part of the maintenance immunosuppressive regimen. Rarely, acute severe hypercalcemia can occur in the immediate post-transplant period, requiring emergency parathyroidectomy. Often these patients were on high doses of cinacalcet prior to transplantation (39). Abrupt discontinuation of cinacalcet post-transplantation coupled with high PTH and vitamin D levels can lead to acute, severe hypercalcemia.

Even though hypercalcemia is multifactorial, most patients have inappropriately high PTH level for the degree of hypercalcemia. In such patients, especially in the first few months after transplant, no further work up may be needed. However, if PTH is appropriately suppressed, non-PTH related causes need to be investigated. Similar to evaluation of hypercalcemia in non-transplant patients, other etiologies including granulomatous disease, milk-alkali syndrome, malignancies need to be ruled out (40–42). Low turnover or adynamic bone disease may be associated with hypercalcemia.

In majority of patients, hypercalcemia is gradual, asymptomatic, and can be medically managed. In patients with mild hypercalcemia, we encourage adequate fluid intake and avoidance of medications that can independently increase serum calcium levels, such as thiazide diuretics and calcium supplements. If vitamin D replete, vitamin D supplements should be discontinued. If hypercalcemia persists despite these measures, and if PTH level is persistently high, cinacalcet can be started temporarily and dose titrated (43). If cinacalcet cannot be continued for financial reasons or intolerance to the drug, then subtotal parathyroidectomy should be considered to treat tertiary hyperparathyroidism (44, 45). Since involution of hyperplastic parathyroid glands and a resultant decline in PTH concentration occur over a year, many transplant physicians prefer to wait for at least a year after kidney transplantation before proceeding to surgery, provided there is no graft dysfunction related to hypercalcemia. Also, treatment of osteoporosis with bisphosphonates and denosumab may improve serum calcium levels.

#### Hypocalcemia

Hypocalcemia is infrequently observed after kidney transplantation. Serum calcium levels may decrease initially in the first week after transplantation, likely secondary to a fall in PTH levels and discontinuation of exogenous calcium and vitamin D supplements (46). In such patients, hypocalcemia is usually mild. However, severe hypocalcemia and hungry bone disease can be seen after parathyroidectomy surgery. While it is hard to predict the severity and duration of hypocalcemia with hungry bone disease, most patients have high pre-surgery PTH levels. Similarly, patients who had undergone parathyroidectomy pre-transplant may be on high dose parenteral vitamin D supplements, and usually the transplant team and the patient are unaware of parenteral medications administered during dialysis. Often these patients are also on calcium-based phosphate binders that are also discontinued post-transplantation. Abrupt cessation of parenteral vitamin D and oral calcium supplements post-transplant may lead to severe hypocalcemia. Lastly, a combination of hyperphosphatemia and associated hypocalcemia can be noted in the immediate post-transplant period in patients with delayed graft function.

### Phosphate disorders

Hyperphosphatemia is usually only seen in patients with delayed graft function, or in transplant patients with advanced CKD.

On the other hand, hypophosphatemia is common in KTRs and occurs in ~50% of patients. It most commonly occurs 3–4 weeks after transplantation, especially in patients with immediate graft function and high pre-transplant PTH levels (47). ESRD patients have high concentrations of PTH and FGF-23 just prior to transplantation. When GFR rises sharply with immediate graft function, these two phosphaturic hormones increase urinary fractional excretion of phosphate and result in significant urinary loss of phosphorus (Figure 1B). It is usually self-limited and serum phosphorus levels begin to normalize within the first few months, correlating with decline in FGF-23 levels (37). However, in a small fraction of patients, renal phosphate wasting persists for few months despite normal phosphate levels, and this may be related to persistent hyperparathyroidism (48).

Among medications, glucocorticoids can reduce the expression of Na-Pi co-transporters and worsen urinary loss of phosphorus (49). Steroids can also reduce oral phosphorus absorption in the intestines (50, 51). Rapamycin may modulate Klotho expression through mTORC2 activation and contribute to hypophosphatemia (52, 53). Tenofovir can cause Fanconi-like syndrome and lead to urinary phosphate loss. In rats, ischemia-reperfusion injury can reduce phosphate reabsorption in the tubules (54). However, it is unclear if phosphaturia is increased in humans who undergo deceased donor transplantation with associated kidney ischemia-reperfusion injury. Recently there have been multiple reports of hypophosphatemia with the use of ferric carboxy-maltose for iron deficiency anemia (55–58) and it appears to be related to acute and reversible increase in FGF-23 levels.

In majority of patients, hypophosphatemia is asymptomatic. Muscle weakness, rhabdomyolysis and hemolysis do not occur until serum phosphorus concentration is <1 mg/dL. Hypophosphatemia may be associated with a lower risk of death-censored graft failure and cardiovascular mortality, but not non-cardiovascular or all-cause mortality (59). On the contrary, another study showed that serum phosphorus <2.5 mg/dL at 1-year post-transplant was associated with higher mortality and death-censored graft failure (60). However, this may likely reflect an underlying association with elevated FGF-23 levels, which has been demonstrated to be a risk factor for graft loss and mortality (61).

### Vitamin D disorders

The majority of KTRs have vitamin D deficiency (62–64). When compared with non-transplant controls, KTRs have significantly lower 25(OH)D levels (65). Even though seasonal variation in serum 25(OH)D concentration due to presence or lack of sun and heat exposure is not consistently observed (63, 66), transplant patients frequently have significant vitamin D deficiency in both seasons. This may be related to avoidance of significant sun exposure and to the use of sunscreen lotions to reduce the risk of skin cancer (62, 63) post-transplant.

From a transplant perspective, low 25(OH)D maybe associated with an increased risk of all-cause mortality. Very low levels may also be associated with rapid decline in kidney function (67). Vitamin D deficiency at 3 months post-transplant may be associated with higher risk for significant interstitial fibrosis and tubular atrophy at 1 year and as a result, lower GFR (68). However, there are conflicting reports about the link of vitamin D deficiency and increased risk of acute rejection (69–72). Also conflicting is the link between low vitamin D levels and increased risk of cytomegalovirus and BK virus infections (71, 73, 74). These observations have not been validated with prospective randomized studies. Results of the randomized trial (VITA-D) to evaluate the effect of cholecalciferol on graft function, acute rejection rates, and the number and severity of infections within first year of transplantation have not yet been published (75).

In the last few decades, vitamin D and its effect on extra-skeletal health has gained significant interest, especially its role in cancer, cardiovascular disease, diabetes, and mortality (76). Among KTRs, studies have not consistently shown an association between low vitamin D levels and risk of cardiovascular diseases or malignancies (77–79). Severe vitamin D deficiency [25(OH)D <10 ng/mL] may be associated with higher risk for post-transplant diabetes (80). A small observational study suggests that the activated vitamin D, paricalcitol, reduces proteinuria in KTRs by reducing inflammation (81). A prospective randomized study (VITALE) underway is evaluating the effect of vitamin D supplementation on a composite endpoint of *de novo* diabetes mellitus, major cardiovascular events, *de novo* cancer and mortality in KTRs (82). Until it sheds light, the link between vitamin D supplementation and extra-skeletal benefits is unknown.

### Hyperparathyroidism

Secondary hyperparathyroidism in advanced CKD results from multiple stimuli, including hyperphosphatemia, hypocalcemia, low 1,25(OH)D levels, and skeletal resistance to PTH. These factors result in continuous stimulation of PTH synthesis and secretion. Parathyroid hyperplasia that ensues is initially diffuse and polyclonal, and still responds to vitamin D therapy and cinacalcet. However, with time, there is down regulation of vitamin d receptors and calcium-sensing receptors in the parathyroid tissue, and hyperplasia often becomes monoclonal or nodular in nature (83). In such patients, PTH synthesis and secretion become autonomous with minimal response to therapeutic agents and is often associated with hypercalcemia. When measured by ultrasound, diffuse polyclonal hyperplastic glands are significantly smaller than nodular monoclonal glands (84).

With successful transplantation and higher GFR, most of these stimuli of parathyroid hyperplasia abate. This often leads to a gradual decline in PTH concentrations. Unlike FGF-23 levels that precipitously decline post-transplant, the fall in PTH is more gradual. It has been reported that 25 to >80% of patients still have inappropriately high PTH beyond 1-year post transplant (37, 85, 86). Pre-transplant cinacalcet use, development of nodular hyperplasia, and dialysis vintage are associated with high PTH levels after transplant (87), while use of vitamin D pre-transplant appears to be protective.

### Renal osteodystrophy

Progressive kidney disease commonly results in the spectrum of bone diseases known as renal osteodystrophy. How this collection of bone diseases evolves in the post-transplant period is unclear due to the lack of available bone biopsies. This paucity of histological evidence hampers our ability to understand the evolution of osteodystrophy post-transplantation. As in the pre-transplant period, vitamin D deficiency has been associated with severe cases of osteomalacia in KTRs. In a small study of 20 subjects, adynamic bone disease was the predominant pathology prior to transplant, and low-turnover disease persisted 6 months after transplant. The relative decrease in PTH secretion as a result of improved phosphaturia may be the reason for these early findings. However, as allograft function deteriorates over time, high-turnover disease, such as osteitis fibrosa cystica, becomes more common (88).

### Osteopenia and osteoporosis

Osteopenia and osteoporosis are conditions highlighted by microarchitectural changes that result in reduced bone mass and increased skeletal fragility. As in the general population, age, race, ethnicity, weight, diabetes mellitus, tobacco use, and menopausal status influence osteoporotic risk. With aging, an imbalance in bone remodeling results from increased osteoclast activity and reduced osteoblast production and differentiation. After transplantation, several other factors including residual MBD, glucocorticoids, hypomagnesemia, and hypogonadism play a role in bone loss.

Transplant medications modify osteoprotegerin and the receptor activator of nuclear factor-κB ligand (RANKL), potent mediators of the bone remodeling process. Through regulation of the RANKL system, glucocorticoids decrease osteoblast proliferation and differentiation, while promoting osteoclastogenesis (89–91). Additionally, they decrease intestinal calcium absorption and increase urinary calcium loss (92). The rate of bone loss is greatest in the first 3–6 months of steroid use when assessed by bone mineral density (BMD) (93). Even when these reductions in bone mass are not evident, low dose glucocorticoid use is still associated with reduced bone strength (89). Microarchitectural changes through increased death of osteocytes are evident regardless of bone volume. In the era of reduced rejection with tacrolimus, strategies to minimize steroid exposure in low immunologic risk recipients can limit glucocorticoid-induced bone loss. However, both tacrolimus and sirolimus can increase osteoblast apoptosis (94).

Hypomagnesemia, possibly by stimulating PTH secretion and osteoclastogenesis while inhibiting osteoblast proliferation, increases fracture risk in dialysis patients (95). Magnesium deficiency is a common complication for KTRs and is often related to medications, including calcineurin inhibitors, proton pump inhibitors, and pentamidine. However, there are no studies to show an independent association between hypomagnesemia and fracture risk in KTRs.

Gonadal hormones play a significant role in achieving peak bone mass, and hypogonadism is associated with bone loss and low BMD. Around 40% of ESRD patients have testosterone deficiency, (96, 97) and sex hormone production can improve significantly within the first 3 months post-transplantation in patients younger than 50 years old (97). Despite resolution of uremia, persistence or development of other chronic diseases and chronic use of glucocorticoids may decrease gonadal hormones in KTRs and promote bone loss.

Other risk factors include poor nutritional status, tobacco use, and alcohol use. Recently, proton pump inhibitors have been associated with hip fractures among KTRs (98). While many mechanisms have been theorized, reduced cation absorption appears to be the leading hypothesis for this mechanism of increased risk.

### Osteonecrosis

Osteonecrosis or avascular bone necrosis, a pathological condition characterized by bone death, has a strong association with glucocorticoid use. Prevalence has been reported to be between 3-40% in different studies (99, 100). However, with introduction of calcineurin inhibitors and consequent reduction in steroid doses, the incidence of osteonecrosis has declined significantly (101). While the exact pathogenesis remains unclear, possible mechanisms include steroid-induced decrease in vascular endothelial growth factor, alterations in circulating lipids with resultant fat emboli, increased apoptosis of osteoblasts, osteocytes and endothelial cells, adipogenesis, procoagulant state, modulation of vasoactive mediators, and elevated intraosseous pressure, which eventually lead to ischemia and necrosis (102). The hips, knees, and shoulders are the most commonly affected sites.

Prevention, early diagnosis, and slowing the progression of osteonecrosis is key, as there is no proven therapy, especially for advanced disease. This includes limiting steroid use, and avoiding other risk factors, including alcohol use and smoking. Surgical options for symptomatic patients with progressive early stage osteonecrosis include core decompression, osteotomy, and bone grafting. When bone collapse has already occurred, hemiarthroplasty or total-hip arthroplasty may be offered. Recently, surgeons have considered combining core decompression with stem cell-based (implantation of autologous bone marrow concentrate or mesenchymal stem cell) and growth factor-based (bone morphogenic proteins, vascular endothelial growth factor) regenerative therapies (103).

## Evaluation of MBD post-transplant

### Biochemical assessment

Biochemical abnormalities of disordered mineral metabolism are common and fluctuate widely, especially in the immediate post-kidney transplant period. Hypophosphatemia occurs early after kidney transplantation. Initial hypocalcemia could be followed by hypercalcemia, and in some patients, hypercalcemia could persist beyond 1-year post-transplantation. Therefore, the KDIGO 2017 guideline update recommends that serum calcium and phosphorus levels be measured at least weekly in the immediate post-kidney transplant period until stable (graded 1B) (104). After the immediate post-kidney transplant period, the frequency of monitoring serum calcium and phosphorus levels can be based on the rate of progression of CKD, the presence and magnitude of abnormalities, and whether the patient is receiving treatments for CKD-MBD (not graded). As shown in Table 2, recommended monitoring intervals include: (1) every 6–12 months in CKD stages 1-3T; (2) every 3–6 months in CKD stage 4T; and (3) every 1–3 months in CKD stage 5T.

**Table 2:** Proposed biochemical testing in kidney transplant recipients.

	**Immediate post-transplant**	**CKD: GFR category**					
		**G1T**	**G2T**	**G3aT**	**G3bT**	**G4T**	**G5T**
Serum calcium	At least weekly	Every 6–12 months 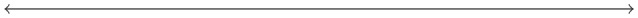				3–6 months	1–3 months
Serum phosphate	At least weekly	Every 6–12 months 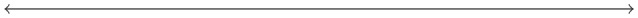				3–6 months	1–3 months
Serum intact PTH	Once	Once* 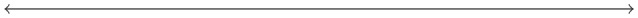				6–12 months	3–6 months
Serum 25(OH)D	Once	Once^#^–correct deficiency and insufficiency 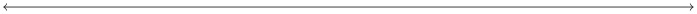					
Bone density		Check to assess fracture risk if risk factors for osteoporosis present 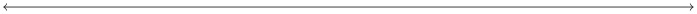					

* *Repeat testing interval determined by baseline value, CKD progression and MBD treatment*.

# *Repeat testing determined by baseline values and interventions*.

Considering the high prevalence of hyperparathyroidism and vitamin D deficiency in KTRs, serum 25(OH)D and PTH levels are commonly measured after transplantation. The KDIGO 2017 guideline update suggests that a baseline 25(OH)D level might be measured in the immediate post-transplant period, and repeated testing should be determined by baseline values and interventions (105). It also recommends measuring a baseline PTH level in the immediate post-transplant period and subsequent monitoring be based on the rate of progression of CKD, baseline level, and whether the patient is receiving treatments for CKD-MBD (not graded). Recommended monitoring intervals include: (1) variable in CKD stages 1-3T, depending on baseline level and CKD progression; (2) every 6–12 months in CKD stage 4T; and (3) every 3–6 months in CKD stage 5T (104, 105). However, there is no recommendation as to what acceptable PTH levels are post-transplant and when intervention is needed. Presumably, the skeletal resistance to PTH is removed with new-found kidney function and loss of uremic toxins, suggesting that levels should substantially reduce or approach the normal range. With poor predictive value and variable degrees of graft function, PTH levels often do not correlate well with bone turnover. Additionally, the levels that are deemed “appropriate” must be individualized to the degree of graft function. Until more specific bone turnover markers that are independent of kidney function can be introduced into regular practice, confidence in treatment exists in the most extreme trends in PTH levels.

## Evaluating fracture risk

### Dual energy X-ray absorptiometry bone mineral density (DXA BMD)

The use of DXA to predict fracture risk in KTRs remains a challenge. Limitations include: (1) inability to distinguish between cortical and trabecular bone, which are differentially affected in secondary hyperparathyroidism; (2) confounding signals from concomitant vascular calcification; and (3) observations that glucocorticoid-induced fractures occur at higher BMD values than in patients with non-glucocorticoid-induced osteoporosis (106). However, a recent study showed that KTRs with hip bone osteopenia (HR 2.7; 95% CI 1.6–4.6) and osteoporosis (HR 3.5, 95% CI 1.8–6.4) noted on DXA have an increased risk of fracture; the predictive value of DXA BMD results in the lumbar spine was much less certain (107). Furthermore, a recent meta-analysis and several prospective studies in adults with CKD G3a to G5D stages showed that DXA BMD, especially at the total hip region, predicts fractures across the spectrum of CKD (108–111). Based on these data, KDIGO guidelines 2017 update recommends BMD testing in patients with CKD G1T-G5T with risk factors for osteoporosis, if results will alter therapy (grade 2C) (105). With the overwhelming majority of KTRs having additional risk factors (steroid use, age, diabetes, etc.) for osteoporosis, screening DXA scans are ordered at months 3 and 15 at our institution.

### Fracture risk assessment tool (FRAX)

The World Health Organization's FRAX Tool is used commonly in the general population to predict the 10-year probability of a major osteoporotic fracture. It utilizes an algorithm that includes age, sex, and several clinical risk factors for fracture, including parental hip fracture, previous fragility fracture, rheumatoid arthritis, current smoking, secondary osteoporosis, low body mass index (BMI < 19 kg/m^2^), prolonged glucocorticoid use, and excessive alcohol intake. The FRAX score does not require bone densitometry data to predict fracture risk, making it an attractive clinical tool. However, the etiology of transplant bone disease is multifactorial, and pathology is widely variable. Therefore, factors in the FRAX algorithm that are associated with fracture risk in the general population may not accurately predict fractures in KTRs.

Recently, two Canadian studies assessed the prognostic value of FRAX in KTRs and patients with reduced kidney function (112, 113). In a single-center, retrospective cohort study of 458 KTRs who were followed for a mean of 6.4 years, the observed 10-year major osteoporotic fracture risk of 6.3% was concordant with FRAX predictions (5.0% with BMD, 5.6% without BMD). Furthermore, FRAX showed modest fracture prediction and discrimination similar to general population (112). The other multicenter, prospective observational study showed that in patients with CKD stages 3-5 (eGFR < 60 ml/min/1.73 m^2^) FRAX was able to accurately predict fracture risk; however, only 4.0% had CKD stage 4 or 5. Moreover, FRAX major osteoporotic fracture discrimination in individuals with eGFR < 60 ml/min/1.73 m^2^ was similar to those with eGFR ≥ 60 ml/min/1.73 m^2^ (113). While these studies show promise, additional validation is needed before FRAX can be used to guide treatment decisions in KTRs.

### Evaluation/imaging beyond DXA

In KTRs, particularly those with reduced kidney function, the presence of renal osteodystrophy complicates our understanding of bone disease. Bone pathology varies between low-turnover, high-turnover, and mixed states. Consequently, 2-dimensional measurements of bone density often provide limited understanding of bone disease, missing measures of quality and strength. Glucocorticoid-induced fractures occurring at relatively higher BMD values (106), substantiates a need for measurements beyond density in KTRs.

High-resolution peripheral quantitative computed tomography provides a mechanism for understanding density and microarchitecture of cortical and trabecular regions separately (114). In particular, cortical porosity may be used as a measure of estimated bone strength. Methods for distinguishing trabecular and cortical compartments are not definitive or standardized. Peripheral examination also ignores the two most common osteoporotic fracture sites: proximal femur and spine. Bone architecture associated with kidney disease is not uniform and radial evaluation may not reflect the turnover, density, and strength present at the hip. Currently, this technology largely exists for research purposes, as the above barriers, along with cost, must be overcome before broader clinical application materializes.

A novel parameter for describing microarchitecture, trabecular bone score, may provide a potential method for examining bone quality and strength through gray-scale variograms of the spine image available from a DXA (115). In KTRs, trabecular bone scores are associated with fractures independent of a Fracture Risk Assessment score including BMD (116). These scores were also predictive of worsening trabecular microarchitecture and failure load as measured by high-resolution peripheral quantitative computed tomography (117) and have already been incorporated into the World Health Organizations FRAX tool. This additional assessment should warrant more attention given its simplicity and accessibility.

### Bone biopsy

Bone biopsy is an informative diagnostic procedure to evaluate bone abnormalities in patients with kidney disease. To interpret bone biopsy results better, TMV classification was developed using three histologic descriptors assessed by bone histomorphometry; bone turnover (T), mineralization (M) and volume (V). While mineralization is classified as normal or abnormal, turnover and volume can be classified as low, normal or high.

Common bone histopathologic descriptions in ESRD patients include normal bone, osteitis fibrosa cystica, mixed uremic osteodystrophy, osteomalacia, and adynamic bone disease. Compared to older studies that showed higher prevalence of mixed uremic osteodystrophy in KTRs (118), recent studies have shown that osteitis fibrosa is more common (88). However, in a more recent study, bone turnover was normal and bone mineralization was delayed in the majority of patients (119).

Typically after kidney transplantation, uremia-related bone changes improve rapidly, even though changes of hyperparathyroidism can take more than a year to improve (120). Dynamic parameters tend to improve in patients with adynamic bone. But in most patients, there is a slight increase in osteoclast function, decrease in osteoblast function, and retardation of dynamic parameters (118). High turnover bone disease improves after transplantation, and low turnover bone disease may emerge (121). During long-term follow up, bone alterations observed consist of a mixed bone disease with features of high bone turnover coexisting with altered bone formation and delayed mineralization (3). The rate of bone formation decreases along with significant lengthening of bone formation period, resulting in reduced amount of bone replaced during a remodeling cycle (122).

Old age, time on dialysis, and time after transplant are significant predictors for negative effect on bone mass (118). Male gender is also a significant predictor, but serum testosterone level does not predict densitometric or histomorphometric variables (123). Among transplant medications, steroids and cyclosporine are associated with osteoclast stimulation and osteoblast suppression and retardation of mineral apposition and bone formation rates (124).

Despite the presence of standardized classification, interpretation of existing literature is difficult. First, only recent studies have used the TMV classification. Second, there was significant variation when bone biopsy was performed after transplantation, making it difficult to interpret. Third, there has been a significant change in immunosuppressive medications over the years, including recent steroid sparing protocols, making it difficult to compare new and old studies. Lastly, since bone biopsy is invasive, most patients had only one biopsy and there is limited data on follow up and evolution of bone histology.

While bone biopsy provides the most direct information regarding bone health, it's relevance is not generalizable to every region. Femoral, lumbar, and radial pathology can be substantially different. More importantly, due to substantial discomfort associated with the procedure, bone biopsies have fallen out of favor. Our institution does not utilize bone biopsy as part of clinical evaluation for the above reasons.

## Treatment

Treatment of MBD post-transplant often requires a holistic approach with ultimate focus on bone health, instead of focusing on individual lab abnormalities. Also, it is important to note that metabolic and bone changes after transplantation tend to be dynamic and a cookie-cutter approach may not be appropriate for all patients.

### Phosphorus, calcium, and vitamin D replacements

#### Phosphate supplementation

Due to ongoing urinary losses in the immediate post-transplant period, it is often difficult to achieve and maintain normal serum phosphate levels with oral replacements. Oral supplements (oral sodium-potassium phosphate tablet and powder) are started when serum phosphate level is <2 mg/dL with a goal to maintain serum level around 2 mg/dL. It is recommended not to elevate serum level to normal range for fear of exacerbating hyperparathyroid state. There is also concern of nephrocalcinosis with aggressive replacement, especially in the presence of hyperparathyroidism, simultaneous use of cinacalcet, and oral alkali. Each 250 mg tablet contains around 8 mmol of phosphate. Neutral phosphate salt supplementation, in addition to correcting hypophosphatemia, has been shown to increase muscle ATP and phosphodiester content without affecting other mineral metabolism and has been shown to improve renal acid excretion (125). In addition to pharmacologic supplementation, we recommend high phosphorus containing diets. We recommend patients to liberalize intake of skim milk every day and other dairy products; 480 mL of skim milk contains 15 mmol of phosphate. If skim milk cannot be tolerated, a less favorable alternative would be temporary intake of diet cola drinks.

#### Calcium and vitamin D supplementation

While most patients with mild hypocalcemia can be managed with oral calcium supplements, patients with hungry-bone disease and severe hypocalcemia need high-dose activated vitamin D such as calcitriol or paricalcitol, along with parenteral calcium infusions followed by high-dose oral calcium supplements. We recommend the use of calcium and cholecalciferol in all kidney transplant patients with normal serum calcium. Especially when steroids are given, administration of vitamin D improves GI calcium absorption. In the absence of randomized trials, the dose and choice of vitamin D is quite variable across transplant centers. Most agree the use of adequate dose of vitamin D to correct vitamin D deficiency and maintain serum 25(OH)D level of >30 ng/mL. The KDIGO 2009 guidelines suggest Vitamin D deficiency should be corrected as recommended for the general population (graded 2C) (104). If the patient develops hypercalcemia, vitamin D supplementation should be discontinued until serum calcium normalizes.

Active vitamin D supplementation has been used successfully to treat secondary hyperparathyroidism in KTRs in much the same way that it is utilized in patients with CKD. In fact, paricalcitol, whether given intravenously or orally, has even been shown to be more effective than cinacalcet in reaching goal PTH and reducing markers of bone turnover (126). Both the use of calcitriol and paricalcitol have resulted in improved BMD while reduced markers of inflammation have been found among KTRs that are administered paricalcitol (127, 128). Paricalcitol has also demonstrated variable results regarding proteinuria reduction in KTRs (128, 129) and has been touted as having an anti-fibrotic advantage over calcitriol in mice studies (127). Despite these benefits, active vitamin D supplementation results in relative increases in FGF-23, the downstream effects of which are still not completely understood.

### Cinacalcet

In KTRs with hyperparathyroidism and hypercalcemia, cinacalcet reduces PTH levels and as a result, improves serum calcium (130–133) and serum phosphate levels (134–136). Regression of parathyroid hyperplasia has been documented (137). Some studies have shown improvement in bone mineral density, especially at the hip level (138, 139). However, these potential benefits must be balanced by the commonly encountered gastrointestinal intolerance and higher urinary fractional excretion of calcium and hypercalciuria (140–144). In fact, reports of allograft nephrocalcinosis (145, 146) and consequent graft failure have been reported. Despite this risk, studies demonstrating no change in allograft function with long-term use of cinacalcet exist (134–136). Due to cinacalcet's effect on renal sodium handling, some studies have shown improvement in blood pressure (141, 147). However, there are no randomized controlled trials that have shown improvement in patient survival, much less fractures.

There may be drug-drug interaction between cinacalcet and tacrolimus; when given together, cinacalcet may reduce tacrolimus concentration (148, 149). While the exact mechanism is unclear (150), it is prudent to monitor tacrolimus levels when cinacalcet is started. This is especially important in sensitized patients who are at high risk for acute rejection. Cinacalcet does not seem to interfere with pharmacokinetics of cyclosporine or mycophenolate (149). It is also important to note that co-administration of a potent CYP3A4 inhibitor (e.g., protease inhibitors, itraconazole, diltiazem etc.) may increase serum levels of cinacalcet since the drug is partially metabolized by that pathway. Serum calcium should be closely monitored in such patients. In addition, cinacalcet is also a potent inhibitor of CYP2D6 pathway. When cinacalcet is combined with other potent inhibitors of CYP2D6 pathway like SSRI antidepressants (fluoxetine, paroxetine), inhibition of this metabolic pathway can be profound. In such patients, doses of medications that are metabolized by CYP2D6 pathway (metoprolol, carvedilol, tricyclic antidepressants, flecainide etc.) should be adjusted to avoid toxicity.

In the post-transplant setting, cinacalcet is primarily used for the management of severe hypercalcemia associated with tertiary hyperparathyroidism. Since cinacalcet is not FDA approved in KTRs, most programs use the drug only in patients with refractory and severe hypercalcemia (corrected serum calcium >11 mg/dL). There are no clear guidelines for the use of this drug. Thus, while the benefit for serum calcium reduction and perhaps BMD improvement is evident in tertiary hyperparathyroidism, its utility in persistent hyperparathyroidism without hypercalcemia or *de novo* hyperparathyroidism is less certain. With unclear targets, particularly as graft function worsens, one risks over-suppression and potential adynamic bone disease.

### Anti-resorptive agents: bisphosphonates

Bisphosphonates are the most commonly used anti-resorptive medication in the general population. However, among KTRs, bisphosphonates have largely demonstrated improvements in BMD without notable changes in fracture risk. Kan et al. conducted a systematic review that noted an improvement solely in lumbar spine BMD (151), while the review performed by Toth-Manikowski et al. demonstrated an association with improved lumbar spine BMD, as well as femoral BMD (152). Unfortunately, proof of improvement in clinical events is lacking from these reviews and trials. Coco and colleagues reported improvements in vertebral BMD, and not hip BMD, among pamidronate recipients, and the limited numbers that received bone biopsies demonstrated a significantly higher number of adynamic bone disease among pamidronate recipients. Thus, this initial improvement in bone volume with bisphosphonates may still portend a substantial fracture risk given the trade-off in bone quality (153). With a very low level of evidence, the KDIGO guideline on transplant suggests considering bisphosphonate treatment within the first 12 months of transplant (154). In particular, the guideline highlights the utility of bone biopsy if considering bisphosphonate therapy. However, excluding adynamic bone disease with this antiquated procedure is not feasible in most transplant centers. Thus, care is reduced to estimating bone turnover rate in conjunction with BMD to determine the utility of bisphosphonates. If the risk of adynamic bone disease can be eliminated with elevated markers of bone turnover such as bone-specific alkaline phosphatase, KTRs with high risk and reduced BMD should be considered for bisphosphonate therapy.

After the first-year post-transplant, insufficient evidence exists to guide whether bisphosphonate therapy should be continued. KTRs should be re-evaluated in terms of their glucocorticoid dose and repeat DXA. Long-term studies examining clinical events are needed to fully understand the utility of bisphosphonate. Additionally, research is needed to understand the utility of alternative therapy in patients with reduced BMD and without overt laboratory evidence of increased bone turnover. More specifically, an alternative anti-resorptive medication, teriparatide, a PTH analogue, may have a role in adynamic bone disease, which is characterized by PTH resistance and relative PTH deficiency.

Denosumab, a RANKL inhibitor presents an attractive alternative, particularly in patients with low GFR. These patients require close monitoring for hypocalcemia. Much like bisphosphonate use, BMD parameters seem to improve, but no evidence exists for changes in fracture risk (155). Teriparatide has been successfully used to improve BMD in patients with glucocorticoid-induced osteoporosis (156). Studies among KTRs are limited by size and have not yielded demonstrable changes in BMD (157). At our institution, when patients present with substantially reduced BMD and elevated markers of bone turnover, we collaborate with colleagues in endocrinology and frequently employ denosumab with concomitant vitamin D therapy.

### Parathyroidectomy

With the advent and expanded therapeutic use of cinacalcet in KTRs, the role of parathyroidectomy (PTX) in tertiary hyperparathyroidism has evolved. In current practice, PTX is limited to KTRs with profoundly elevated parathyroid hormone and calcium levels, symptomatic disease (fractures, EKG changes, neurologic sequalae, etc.) or failure of long-term medical management (158, 159).

When compared with cinacalcet, a greater percentage of KTRs who underwent subtotal parathyroidectomy had normocalcemia, normal PTH levels, and increased femoral neck bone mineral density at 1 year (160). However, the applicability of data is limited by the small sample size, lack of long-term analysis and the patterns of cinacalcet dose adjustments in the study population (161). Further, in clinical practice, the choice of intervention is undoubtedly influenced by multiple factors, including patient preference, patient suitability as an operative candidate, access to therapy, and potential financial costs.

The optimal timing and choice of surgery for tertiary hyperparathyroidism is often debated. Reduced all-cause and cardiovascular mortality is seen among patients with CKD and severe secondary hyperparathyroidism, and presumably pre-transplant surgery offers this benefit among KTRs (162). Among patients with secondary hyperparathyroidism, the inability to determine which glands will involute with new-found kidney function and medical therapy clouds the determination of pre-transplant PTX utility. Consequently, we are reliant on PTH levels and nuclear imaging. As previously mentioned, larger, more nodular glands are likely to be monoclonal and therefore have persistent secretion post-transplant. The optimal level at which PTX confers an advantage in secondary hyperparathyroidism remain unclear. A large retrospective examination of the SRTR of 11,776 patients did not show any association between pre-transplant PTH values greater than 800 pg/mL and graft failure, delayed graft failure, or acute rejection (163). On the other hand, a more recent retrospective study of 913 patients at a single center that underwent kidney transplantation concluded that levels greater than 6 times normal were associated with graft failure, and pre-transplant PTX decreased the risk of allograft failure (164). Thus, we believe that pre-transplant PTX should be determined on a case by case basis. Extreme values with evidence of nodularity and significantly increased size may warrant surgical intervention prior to transplant to prevent further complications in the post-transplant period.

Even though high PTH levels (>500 pg/mL) and serum calcium level (>9.5 mg/dL) are risk factors for needed PTX post-transplant, enough time should be given for spontaneous regression of gland hyperplasia if surgery is planned post-transplant. Post-transplant PTH levels appear to fall rapidly in functional allografts 3-6 months post-operatively, and then follow a more gradual decline that plateaus at 1 year (158, 159). It follows then that referral for operative intervention for persistent disease should be delayed at least 3 months, but not extend past 1 year due to cumulative risk of cardiac, bone and graft complications associated with high PTH and calcium levels (158). Data regarding the effect of parathyroidectomy on renal allograft function is conflicting, with some studies suggesting decreasing function post intervention, while others found no significant long-term difference (44, 158, 165).

The choice of operation has evolved over time. Classic approaches included subtotal or total PTX with auto transplantation (45, 158). Current reports suggest a less extreme approach, including less-than-subtotal parathyroidectomy, may achieve similar therapeutic goals (158, 166). Known complications following PTX include recurrent laryngeal nerve injury (2%), wound bleeding (0.3%), and transient symptomatic hypocalcemia (20–85%).

## Conclusions

The risk of bone disease and its complications vary throughout the post-transplant timeline. Appreciating the residual maladaptive responses found in end-stage kidney disease and the new onset risks that accompany transplant are key to perioperative management. As most MBD parameters begin to normalize with time, subtle aspects of evaluation must be appreciated to reduce the development of bone pathology. Clinicians encounter a different spectrum of MBD abnormalities including hypophosphatemia, hypercalcemia, and persistent hyperparathyroidism. Reversal of MBD abnormalities and vitamin D deficiency is the focus of initial management of KTRs. Parathyroidectomy is reserved for patients with tertiary hyperparathyroidism who fail medical treatment. Later in the post-transplant period, clinicians should aim to mitigate age-related bone disease. While bisphosphonates demonstrate improvements in bone mineral density, no evidence exists for impacting fracture risk and more novel therapies require further study.

Limitations to evaluation center around the poor predictive value of laboratory measures, imaging, and clinical tools. PTH values are idiosyncratic in their influence by graft function and pre-existing nodular hyperplasia of the parathyroid gland. The addition of trabecular bone score may be able to capture measures of bone quality and strength that are missing from DXA scans alone. The FRAX tool shows early promise in its prediction of fractures among KTRs.

The heterogeneity of KTRs and competing influences make studying therapies directed at MBD particularly challenging. Additionally, the latent period before detectable clinical events, such as fractures, is lengthy. With more detailed and specific surrogate markers, investigators will be able to generate feasible studies with increased clinical relevance.

## Author contributions

CV, JP, RC and VR actively contributed to the manuscript by writing individual sections of the final paper. As per the International Committee of Medical Journal Editors guidelines, all authors qualify for authorship of this manuscript.

### Conflict of interest statement

The authors declare that the research was conducted in the absence of any commercial or financial relationships that could be construed as a potential conflict of interest.
